# Sex Differences in Cardiac Pathology of SARS-CoV2 Infected and *Trypanosoma cruzi* Co-infected Mice

**DOI:** 10.3389/fcvm.2022.783974

**Published:** 2022-03-11

**Authors:** Dhanya Dhanyalayam, Hariprasad Thangavel, Kezia Lizardo, Neelam Oswal, Enriko Dolgov, David S. Perlin, Jyothi F. Nagajyothi

**Affiliations:** Center for Discovery and Innovation, Hackensack Meridian Health, Nutley, NJ, United States

**Keywords:** Chagas' heart disease, CoV2 infection, inflammation, cardiomyopathy, adiponectin, mitochondrial oxidation, energy metabolism, glycolysis

## Abstract

Coronavirus disease-2019 (COVID-19) caused by Severe Acute Respiratory Syndrome Coronavirus 2 (SARS-CoV-2; CoV2) is a deadly contagious infectious disease. For those who survive COVID-19, post-COVID cardiac damage greatly increases the risk of cardiomyopathy and heart failure. Currently, the number of COVID-related cases are increasing in Latin America, where a major COVID comorbidity is Chagas' heart disease, which is caused by the parasite *Trypanosoma cruzi*. However, the interplay between indeterminate Chagas disease and COVID-19 is unknown. We investigated the effect of CoV2 infection on heart pathology in *T. cruzi* infected mice (coinfected with CoV2 during the indeterminate stage of *T. cruzi* infection). We used transgenic human angiotensin-converting enzyme 2 (huACE2/hACE2) mice infected with CoV2, *T. cruzi*, or coinfected with both in this study. We found that the viral load in the hearts of coinfected mice is lower compared to the hearts of mice infected with CoV2 alone. We demonstrated that CoV2 infection significantly alters cardiac immune and energy signaling via adiponectin (C-ApN) and AMP-activated protein kinase (AMPK) signaling. Our studies also showed that increased β-adrenergic receptor (b-AR) and peroxisome proliferator-activated receptors (PPARs) play a major role in shifting the energy balance in the hearts of coinfected female mice from glycolysis to mitochondrial β-oxidation. Our findings suggest that cardiac metabolic signaling may differently regulate the pathogenesis of Chagas cardiomyopathy (CCM) in coinfected mice. We conclude that the C-ApN/AMPK and b-AR/PPAR downstream signaling may play major roles in determining the progression, severity, and phenotype of CCM and heart failure in the context of COVID.

## Introduction

COVID-19 illness, caused by severe acute respiratory syndrome coronavirus 2 (SARS-CoV-2; CoV2), results in debilitating disease manifestations in many infected people and increases mortality in people with comorbidities, including heart disease (1–6). The causes of death in COVID-19 patients include cardiomyopathy, stroke, cardiac arrest, sepsis, and organ failure (7–10). Furthermore, post-COVID patients exhibit various degrees of cardiac damage, which may cause debilitating long-term effects on heart function (11–13). Thus, the post-COVID effect may pose a major threat for the development of cardiomyopathy and heart failure, especially in individuals with pre-existing heart conditions.

Although currently deaths due to COVID-19 are subsiding in many countries due to vaccination (14), COVID-19 is still a major threat in Latin America, where a major COVID-19 comorbidity is Chagas Disease (CD). CD is caused by the parasite *Trypanosoma cruzi*, which infects an estimated eight million people in Latin America and is also increasingly found in non-endemic countries, including 300,000 infected individuals in the United States (15). Of these chronically infected individuals, 30% will develop chronic Chagas cardiomyopathy (CCM) and congestive heart failure, which are significant causes of morbidity and mortality (16). Thus, vulnerable COVID-19 patients with CD constitute a major health burden in the Americas. In addition, the post-COVID effect on CCM in CD patients could create a health crisis in Latin America during the post-COVID era since hundreds of thousands of asymptomatic (indeterminate) CD patients likely already have or will contract COVID-19. A recent clinical registry data study from Brazil suggests that Chagas disease and SARS-CoV-2 coinfection do not lead to worse in-hospital outcomes (17). In another case study, the authors reported that COVID-19 infected CD patients (*n* = 2) presented with a rapid disease progression, and despite all efforts of the medical team, both patients died (18). These authors also suggested that COVID-19 may lead to lymphopenia, which could curb the anti–*T. cruzi* immune response and increase the risk of death in coinfected patients (18). However, there is limited clinical data or information from animal models on the interplay between indeterminate/asymptomatic CD and COVID susceptibility, severity, risk of mortality, and long-term effects on heart pathology in post-COVID CD patients.

Recent clinical meta-analysis data for COVID-19 suggest that male sex is independently associated with hospitalization, ICU admissions, need for vasopressors or endotracheal intubation and mortality (19). Many clinical studies have also reported that males have a higher mortality rate due to Chagas' heart disease (20, 21). Male CD patients are also at higher risk for myocardial fibrosis and more severe ventricular remodeling (21). However, the role of sex differences in the interactions between COVID and CD is unknown.

In the present pilot study, we investigated the effect of CoV2 infection on heart metabolism and pathogenesis in mice with indeterminate stage *T. cruzi* infection. We used huACE2 mice (male and female) infected with CoV2, *T. cruzi*, or coinfected with both. Our results show that the pulmonary pathology in coinfected male mice was significantly reduced compared to CoV2 infected male mice and the viral load in the lungs of coinfected mice was reduced compared to CoV2 infected mice. We also show the presence of CoV2 in the hearts of infected mice and that the viral load was significantly reduced in the lungs of coinfected mice compared to mice infected with CoV2 alone. Our data show no difference in heart viral loads between the male and female coinfected mice. However, our data demonstrate a significant difference in the effect of CoV2 infection on cardiac adipogenic metabolism, inflammation, energy metabolism, and mitochondrial functions between male and female coinfected mice compared to their respective control groups. Our data suggest that adiponectin-AMP-activated protein kinase (C-ApN-AMPK) signaling and peroxisome proliferator-activated receptor (PPARγ and PPARα) signaling dominates in the hearts of coinfected mice. At the same time, high level β-adrenergic receptor (b-AR) activity in the hearts of female coinfected mice shifts the energy metabolic pathways toward lipid β-oxidation pathway and is likely responsible for sex differences in the pathogenesis of post-COVID dilated cardiomyopathy, cardiac atrophy and heart failure.

## Materials and Methods

### Biosafety

All aspects of this study were approved by the Institutional Animal Care and Use and Institutional Biosafety Committee of Center for Discovery and Innovation of Hackensack University Medical Center (IACUC 282) and adhere to the National Research Council guidelines.

### Animal Model and Experimental Design

The transgenic mice expressing the human angiotensin-converting enzyme 2 (huACE2) (Jackson Laboratories, Bar Harbor, ME) were bred at Hackensack Meridian Health - Center for Discovery and Innovation (CDI). The Brazil strain of *T. cruzi* was maintained by passage in C3H/HeJ mice (Jackson Laboratories, Bar Harbor, ME). Both male and female mice (*N* = 16) were intraperitoneally (i.p.) infected with 10^3^ trypomastigotes at 6 weeks of age. Mice were maintained on a 12-h light/dark cycles and housed in groups of 3–5 per cage with unlimited access to water and chow. Once they reached indeterminate stage (22) (65 DPI; no circulating parasitemia and pro-inflammatory markers), one set of mice was coinfected intra-nasally with 1 x 10^4^ pfu SARS-CoV2 (NR-52281, Isolate USA-WA1/2020 COV-2 virus, NIH-BEI resources). After 10 DPI CoV2 (i.e., 75 DPI *T. cruzi* infection), we collected samples (heart, lungs, white adipose tissue (WAT) and blood; *n* = 4/sex/subset). Age and sex matched huACE2 mice infected with SARS-CoV2 alone, as well as uninfected huACE2 mice, served as controls (Supplementary Figure 1). The heart samples were used in the present study.

### Immunoblot Analysis

Tissue lysates were prepared as previously described (22). Each sample containing 30 μg of protein were resolved on SDS-PAGE and separately on native gel electrophoresis and the proteins were transferred to nitrocellulose membrane for immunoblot analysis. Adiponectin-specific mouse monoclonal antibody (#ab22554, Abcam), AdipoR1-specific rabbit polyclonal antibody (#ab70362, Abcam), AdipoR2-specific rabbit polyclonal antibody (#ABT12, Sigma-Aldrich), PPARα-specific rabbit polyclonal antibody (#PA1-822A, Thermo Fisher Scientific), PPARγ-specific rabbit polyclonal antibody (#2492, Cell Signaling Technology), pAMPK-specific rabbit monoclonal antibody (#2535S, Cell Signaling Technology), Cytochrome C-specific rabbit monoclonal antibody (#4280S, Cell Signaling Technology), Superoxide dismutase 1-specific mouse monoclonal antibody (#4266S, Cell Signaling Technology), Hexokinase 2-specific rabbit monoclonal antibody (#2867S, Cell Signaling Technology), β1 adrenergic receptor-specific rabbit polyclonal antibody (#12271S, Cell Signaling Technology), F4/80-specific rat monoclonal antibody (#NB 600-404, Novus Biologicals), TNFα-specific rabbit polyclonal antibody (#ab6671, Abcam), pHSL (Ser563)-specific rabbit monoclonal antibody(#4139, Cell Signaling Technology), ATGL-specific rabbit monoclonal antibody(#30A4, Cell Signaling Technology, Perilipin-specific rabbit monoclonal antibody (#D1D8, Cell Signaling Technology), IFNγ-specific rabbit monoclonal antibody (#EPR1108, Abcam), CD4-specific rabbit polyclonal antibody (#NBP1-19371, Novus biologicals), CD8-specific rabbit polyclonal antibody(#NBP2-29475, Novus biologicals), T-cadherin-specific rabbit polyclonal antibody (#ABT121, Millipore), FABP4-specific rabbit monoclonal antibody (#3544, Cell Signaling Technology), IL6-specific mouse monoclonal antibody (#66146-1-lg, Proteintech), IL10-specific rabbit polyclonal antibody (#20850-1-AP, Proteintech), BNIP3-specific rabbit monoclonal antibody (#44060, Cell Signaling Technology), Caspase 3-specific rabbit polyclonal antibody (#9662, Cell Signaling Technology) were used as primary antibodies. Horseradish peroxidase (HRP)-conjugated anti-mouse immunoglobulin (#7076, Cell Signaling Technology) or HRP-conjugated anti-rabbit immunoglobulin (#7074, Cell Signaling Technology) antibody was used to detect specific protein bands (as shown in the figure legends) using a chemiluminescence system. β-actin-specific rabbit monoclonal antibody (#4970S, Cell Signaling Technology) or Guanosine nucleotide dissociation inhibitor (GDI) (#71-0300, Invitrogen) were used as protein loading controls.

### Determination of Parasite (*T. cruzi*) Load in the Tissue

A quantitative real time polymerase chain reaction (q-RT-PCR) was used to quantify the parasite load by using PCR SYBR Green Master Mix (Roche, Applied Science, CT) containing MgCl_2_ by employing QuantStudio 3 Real Time PCR system (Thermo Fisher). DNA isolation, preparation of standard curve and qPCR analysis was performed as previously published (23).

### Determination of SARS-CoV-2 Load in the Tissue

Total RNA was isolated from the hearts using Trizol reagent. The number of SARS-COV-2 copies were quantified using 2019-nCoV_N2 primer/probe mix and One-Step PrimeScript RT-PCR kit from Takara Bio Inc. All assays were performed on Agilent AriaMx Real-time PCR System according to the following cycling conditions: 15 min at 42 °C (1 cycle, reverse transcription), followed by 10 sec at 95 °C (1 cycle, hot start) and continuing with 5 s at 95 °C, and 30 s at 55°C (40 cycles, PCR amplification).

### Histological and Morphometric Analysis of the Heart

The hearts were harvested immediately after sacrificing the mice. The hearts were cut 5 mm above the apex in cross section through the ventricles, fixed in formaldehyde, analyzed by histological staining as described earlier (24). Hematoxylin and eosin (H&E) and Masson's trichrome staining were performed, and the images were captured and analyzed as previously described (25). Four to six sections of each heart were scored blindly. For each myocardial sample, histologic evidence of myocarditis and inflammation was classified in terms of degree of infiltration of immune cells, fibrosis and accumulation of lipid droplets in capillaries was graded on a five-point scale ranging from 0 to 4+. A zero-score indicated lowest or negligible changes and 4 the most damaged state. The cardiomyocyte cell size in the heart sections was analyzed by counting the number of cardiomyocyte nucleus/5 images/heart section (40 x images of H&E stained heart sections). We performed morphometric analysis as described earlier (24). Briefly, the H&E sections of the hearts were used to analyze the thickness of the left ventricular wall (LVW), right ventricular wall (RVW) and the intra-septal wall (24). The thickness of the LVW, RVW and septal wall was measured at five different locations at a magnification of 10x (24). The average value of the 5 measurements was calculated for each mouse.

#### Right Ventricle Hypertrophy

Myocyte profiles in cross section were selected for the analysis of myocyte size adjacent to the inner wall of the right ventricles from the microscopic images of Masson-Trichrome sections (Supplementary Figure 3). The cardiomyocytes have a prolate spheroid shape. Cardiomyocyte length (Dmaj) and diameter (Dmin) were measured on digitized images of tissue slices stained with trichrome stain as reported earlier (Supplementary Figure 3C) (26, 27). Each cell was recognized based on the intercellular collagen network. A total of 40 cells/sex/group were measured and the mean of the cell volume was calculated using the web-based tool [https://www.easycalculation.com/shapes/surface-area-of-prolate-spheroid.php].

### Histological Analysis of the Lungs

Freshly isolated lung tissues were fixed with 10% neutral-buffered formalin for a minimum of 48 h and then embedded in paraffin wax (*n* = 4/sex). Hematoxylin and eosin (H&E) staining was performed, and the images were captured as previously published (25). Four to six sections of each lung were scored blindly. For each lung sections, the histological evidence of pulmonary pathology was classified in terms of the presence of infiltration of immune cells, granulomas, accumulation of lipid droplets and fibrosis as published earlier (23, 25).

### Immunohistochemical Analysis of Perilipin and Phospho-Perilipin

Freshly isolated heart tissues were fixed with 10% neutral-buffered formalin for a minimum of 48 h and then embedded in paraffin wax (*n* = 4/sex) and sectioned for immunohistochemical analysis (IHC). IHC was performed using perilipin and phospho-perilipin specific antibody with a dilution of 1:100 followed by corresponding HRP-conjugated goat anti-rabbit or anti-mouse immunoglobulin as previously described (23, 25). The positive staining intensities of the images were quantified using NIH-Image J software for a minimum of 5 images of each heart (23).

### Statistical Analysis

Statistical analysis was performed using GraphPad Prism (GraphPad Software, Inc., La Jolla, CA, USA). We performed statistical analysis by comparing the data between the infected groups (CoV2/*T. cruzi*/coinfection) and uninfected control groups. For the coinfection group, since the baseline is mice infected with *T. cruzi*, we also performed statistical data analysis and fold change analysis by comparing the data between the coinfected groups and *T. cruzi* infected groups. Comparisons between groups were made using Two-Way ANOVA (GraphPad) and unpaired Student's *t*-test (Microsoft Excel) as appropriate. Values of *p* < 0.05 were considered statistically significant. Data represent means ± S.E.M.

## Results

We developed indeterminate CD model by infecting one set of hACE2 mice with a low dose (1000 parasite) of *Trypanosoma cruzi* (22). At 65 DPI, *T. cruzi* infected mice showed no parasitemia or significant changes in inflammatory markers in blood (data not shown). We analyzed the parasite load in the hearts of *T. cruzi* infected and coinfected mice by qPCR and detected 1.5–3 pg of *T. cruzi* DNA/ng of host DNA (23). We found no significant difference in the parasite load in the hearts between male and female, and *T. cruzi* infected and coinfected mice. To investigate the pathological effects of CoV2 infection on the hearts in *T. cruzi* compromised mice, we performed histological and biochemical analyses of heart and lung samples obtained from the following three different murine models of infections: the *T. cruzi* model (infected with *T. cruzi*), the CoV2 model (infected with SARS-CoV2), and the coinfection model [infected with *T. cruzi* followed by SARS-CoV2 infection at the indeterminate stage (65DPI)]. Uninfected mice served as controls. We used *n* = 8 mice/group for both sexes. We observed no mortality during CoV2 infection in mice with or without *T. cruzi* infection. During the histological analysis of the lung and heart samples, we observed a significant difference in their pathology between the sexes. Therefore, we analyzed all the data separately for males and females in each group as presented below.

### Asymptomatic *T. cruzi* Infection Modulates Pulmonary Pathology During CoV2 Infection in huACE2 Mice

**Figure 1:**
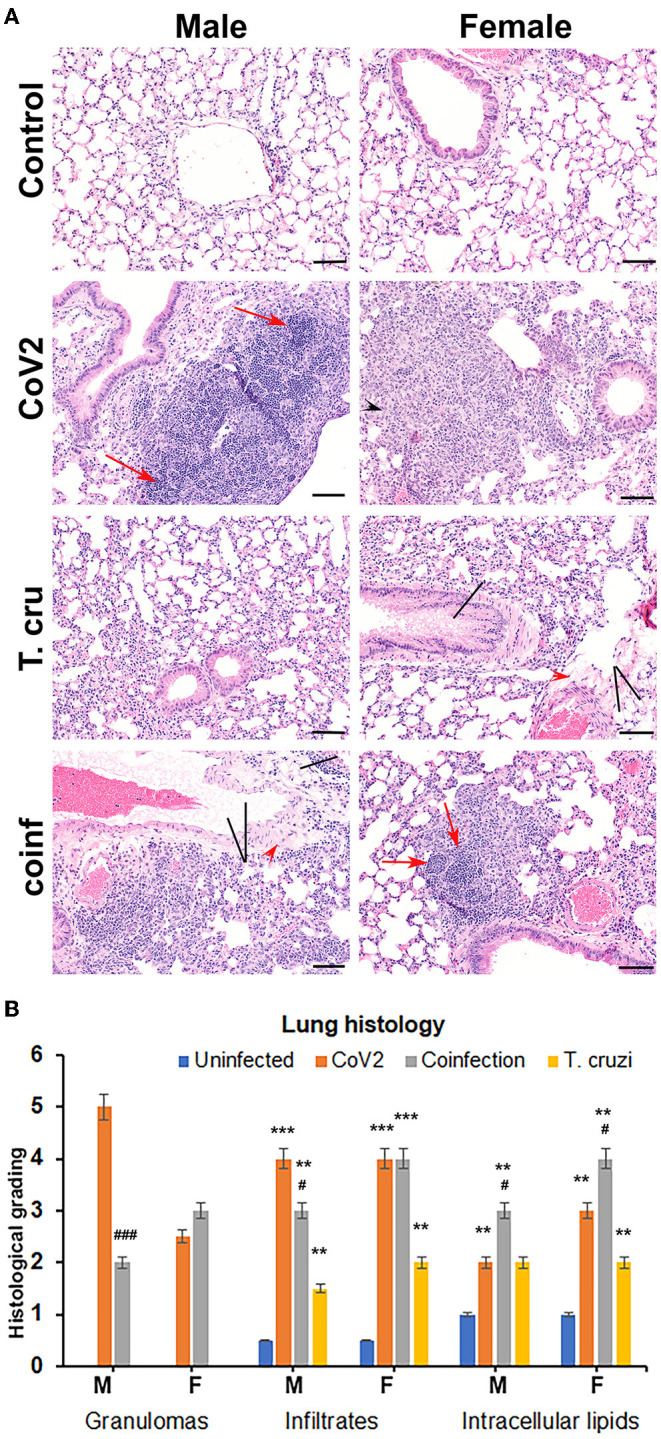
*T. cruzi* infection modulates pulmonary pathology in CoV2 infected huACE2 mice. **(A)** H&E stained lung sections (*n* = 4/sex, a minimum of five images/section were analyzed) of both male and female uninfected, CoV2 infected, *T. cruzi* infected, and coinfected huACE2 mice showing infiltrated immune cells (black arrowhead), accumulated lipid droplets (black line), fibrosis (red arrowhead), and granulomas (red arrow) (20x magnification, bar-200μm). **(B)** Histological grading of lung pathology was carried out according to experimental groups and classified in terms of granulomas (numbers), infiltrated immune cells, and accumulated lipid droplets. Each class was graded on a 6-point scale ranging from 0 to 5 as discussed in Materials and Methods. Values plotted are mean ± standard error (SE) from *n* = 5. The error bars represent the standard error of the mean. **P < 0.01 and ***P < 0.001, between the indicated groups and uninfected mice. ^#^*p* ≤ 0.05 and ^###^*p* ≤ 0.001 for comparisons between CoV2 infected and co-infected mice.

We and others have shown that *T. cruzi* infection alters immune and metabolic signaling in mice during acute and chronic stages (28, 29). Here, we analyzed whether asymptomatic (indeterminate stage) *T. cruzi* infection-induced immune and metabolic changes regulate pulmonary pathology in intranasally CoV2 infected huACE2 mice 10 days post-CoV2 infection. Histological analysis of H&E stained lung sections of uninfected (control), CoV2 infected (positive control), *T. cruzi* infected, and coinfected mice were analyzed for infiltrated immune cells, accumulated lipid droplets, fibrosis, and granulomas (Figure 1A). Histological analysis showed significantly increased infiltrated immune cells and lipid droplets in the lungs of *T. cruzi* infected mice compared to uninfected mice (Figures 1A,B). The alveolar space was more constrained and interstitial tissue thickened in male *T. cruzi* infected mice compared to female T. *cruzi* mice. CoV2 infection also significantly increased infiltration of immune cells and lipid droplets in the lungs compared to uninfected mice (sex and age matched). However, the number of granulomas and their size were greater in male CoV2 mice compared to female CoV2 mice. Interestingly, both the number and size of granulomas were greater in the lungs of female coinfected mice than in male coinfected mice. For both sexes, we observed vascular leakage (hemosiderin) and neutrophilic alveolitis in the lungs in CoV2/coinfected mice. These analyses demonstrated that: (i) the pulmonary pathology in coinfection is reduced compared to CoV2 infection alone; and (ii) although males are more susceptible to severe pulmonary CoV2 infection in general, in the context of *T. cruzi* coinfection females are more susceptible to severe pulmonary CoV2 infection compared to males.

### Asymptomatic *T. cruzi* Infection Reduces Viral Burden in the Lungs of huACE2 Mice Coinfected With SARS-CoV2

**Figure 2:**
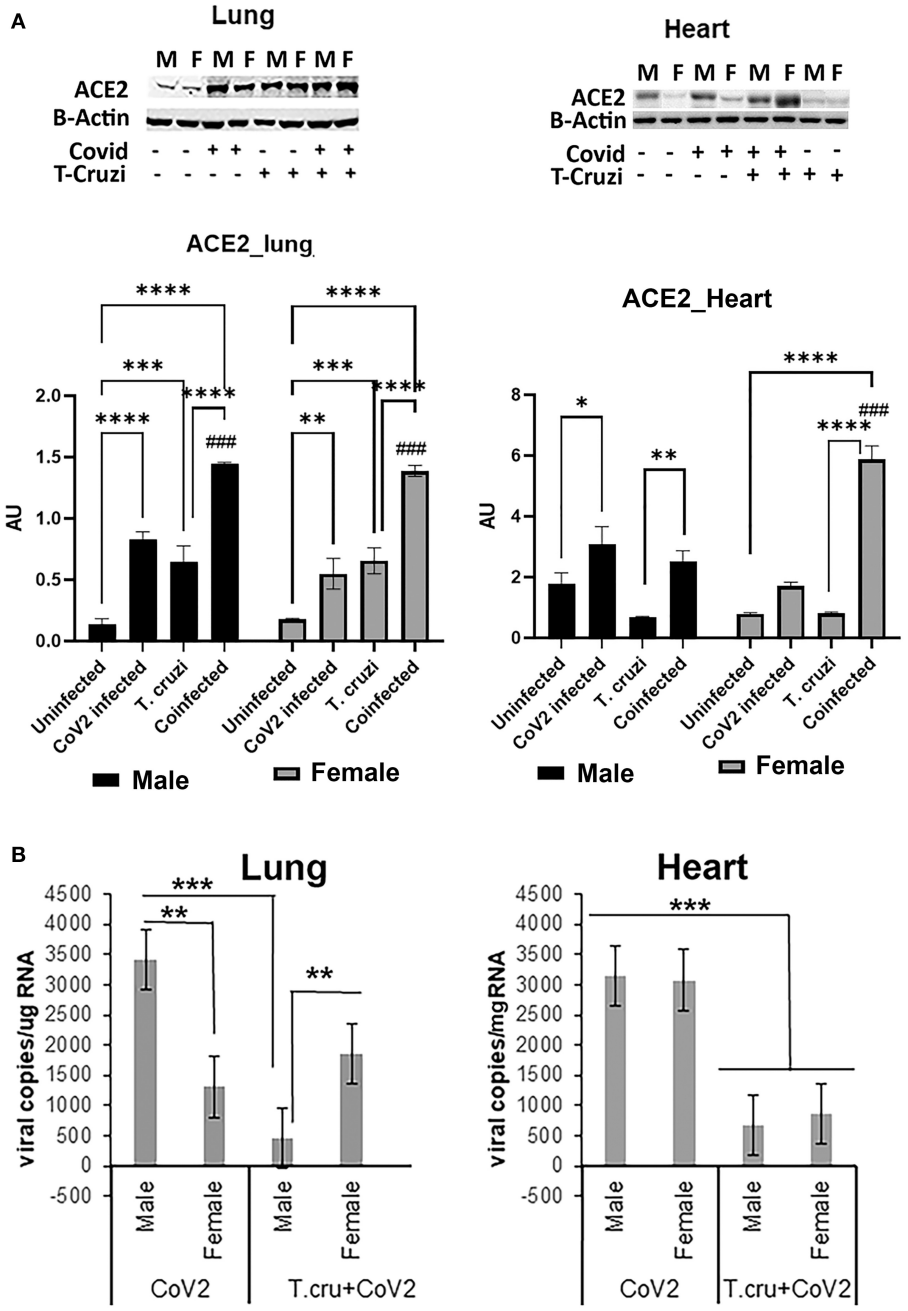
Changes in ACE2 levels and viral load in the lungs and hearts of CoV2 infected, *T. cruzi* infected, and coinfected hACE2 mice. **(A)** Immunoblot analysis (upper panel) of ACE2 in the lungs (left) and hearts (right). GDI was used as loading control. Fold changes in the protein levels of ACE2 were normalized to GDI expression and are shown as a bar graph (A dot plot displaying individual data point is shown in Supplementary Figure 4). The error bars represent standard error of the mean. *p < 0.05, **p < 0.01, ***p < 0.001 and ****p < 0.0001 compared to uninfected sex matched mice (*n* = 4/sex/group) (^*###*^*p* < 0.001 for comparisons between CoV2 infected and coinfected mice). **(B)** Number of viral copies/μg of RNA in the lungs (left) and hearts (right) quantitated by qPCR in male and female CoV2 and coinfected mice. The error bars represent standard error of the mean (**p ≤ 0.01 and ***p ≤ 0.001) (M, male; F, female).

ACE2 is a known receptor for the cell entry of SARS-CoV2 (30, 31). It has been shown that ACE2 expression levels were positively associated with immune signatures and no significant difference in their levels between males and females or between younger and older persons in any tissue (32). Since *T. cruzi* infection can alter the immune signature, we analyzed the effect of *T. cruzi* infection on the expression levels of ACE2 in the lungs and hearts by Western blotting (Figure 2A). As expected, CoV2 infection significantly increased ACE2 levels in the lungs in huACE2 mice in both males (*p* ≤ 0.0001) and females (*p* ≤ 0.01) compared to uninfected sex-matched mice (Figure 2A). The levels of ACE2 were significantly higher (*p* ≤ 0.001) in the lungs of both male and female *T.cruzi* infected mice compared to sex matched control mice (Figure 2A). CoV2 infection further significantly increased (*p* ≤ 0.01) the levels of ACE2 in the lungs of both males and females in coinfected mice (Figure 2A). Our data showed a significant increase in ACE2 levels in the lungs of coinfected mice compared to only CoV2 infected mice (Figure 2A). We observed no difference in the levels of ACE2 in the lungs between the sexes in coinfected mice. In the hearts, in uninfected female mice, the levels of ACE2 were significantly lower (*p* ≤ 0.01) compared to uninfected male mice (Figure 2A). CoV2 infection significantly increased ACE2 levels in the hearts of male (*p* ≤ 0.05) mice. *T. cruzi* infection did not significantly alter the levels of ACE2 in the hearts of male and female mice compared to sex matched uninfected control mice. ACE2 levels in the hearts significantly increased in coinfected male (*p* ≤ 0.01) and coinfected female (*p* ≤ 0.0001) mice compared to sex matched *T. cruzi* infected mice, and significantly increased only in coinfected female mice (*p* ≤ 0.0001) compared to sex matched uninfected mice. These data suggest that ACE2 is highly expressed in the lungs of coinfected male and female mice and in the hearts of coinfected female mice compared to CoV2 infected (male/female) mice.

Lung viral loads quantitated by qPCR analysis were significantly greater (*p* ≤ 0.01) in male CoV2 infected mice compared to female CoV2 infected mice (Figure 2B). Interestingly, although ACE2 levels were significantly higher in the lungs of male coinfected mice compared to male CoV2 infected mice (Figure 2A), the viral load in the lungs of male coinfected mice was significantly lower (*p* ≤ 0.001) compared to male CoV2 mice (Figure 2B). Between coinfected males and females, the viral load in the lungs of female mice was significantly higher (*p* ≤ 0.01) compared to male mice (Figure 2B). However, the viral load in the lungs of coinfected female mice was not significantly altered compared to CoV2 infected female mice. These data suggest that males are likely more susceptible to pulmonary CoV2 infection in general, but that females may be more susceptible to pulmonary CoV2 infection in the context of CD. ACE2 levels were either similar (in males) or significantly greater (in females) in coinfected mice compared to CoV2 infected male or female mice (Figure 2A). However, the viral load was significantly lower in the hearts of coinfected male and female mice (4.7-fold and 3.6-fold, respectively, *p* ≤ 0.001) compared to CoV2 infected male and female mice (Figure 2B). These data suggest that: (i) CoV2 infects myocardium in huACE2 mice infected intranasally with SARS-CoV2 and (ii) indeterminate stage *T. cruzi* infection reduces the viral load in the heart during CoV2 infection.

### Sex Dependent Morphological Changes in the Hearts of Mice Infected With CoV2, *T. cruzi*, and Coinfection

**Figure 3:**
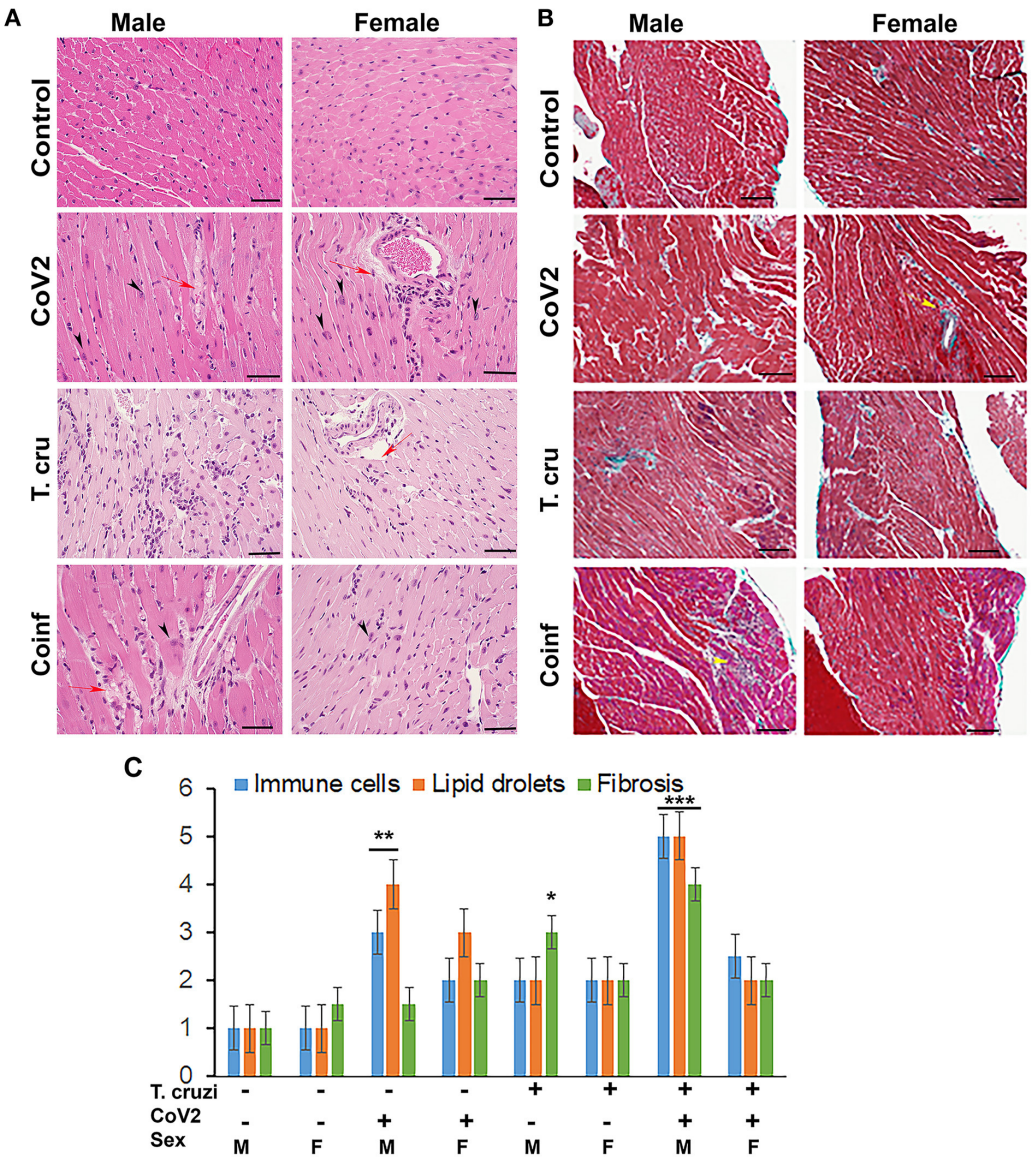
Histology of the myocardium of uninfected, CoV2 infected, *T. cruzi* infected, and coinfected huACE2 mice (*n* = 4/sex, a minimum of five images/section were analyzed). **(A)** H&E stained sections of left ventricles showing the accumulation of lipid droplets in the capillaries (red arrow) and presence of enlarged cardiomyocyte nuclei (black arrowhead) in infected mice (20x magnification). **(B)** Masson-trichrome stained sections of right ventricles showing fibrosis (blue and purple) and the presence of immune cells (yellow arrows) in the right ventricles (RV) of infected/coinfected mice (20x magnification; bar – 100 μm) (Additional images are shown in Supplementary Figures 2, 3). **(C)** Histological grading of heart sections showing the levels of immune infiltrates, lipid droplet accumulation, and fibrosis among CoV2 infected, *T. cruzi* infected, and coinfected mice compared to uninfected mice in both sexes. The error bars represent standard error of the mean. *p < 0.05, **p < 0.01 and ***p < 0.001 compared with uninfected sex matched mice (M, male; F, female).

We have shown that CoV2 infects and persists in the hearts of intra-nasally infected mice (Figure 2B). Histological analysis of the hearts was performed using H&E (Figure 3A) and Masson-trichrome (Figure 3B) stained sections as described in Materials and Methods. Microscopic analysis of the heart sections of CoV2 infected mice demonstrated the presence of infiltrated immune cells, increased accumulation of lipid droplets in the capillaries, enlarged cardiomyocyte nuclei, and increased fibrosis compared to control mice (Figures 3A,B and Supplementary Figure 2). The H&E sections showed significantly reduced cytoplasmic coloration in LV of female coinfected mice compared to their sex matched counterparts (Figure 3A), which is an indication of reduced intracellular protein levels. H&E sections of the hearts showed the presence of myocytes with significantly increased cell size in LV in coinfected male mice compared to coinfected female mice and also compared to uninfected mice (Supplementary Figure 3 and Supplementary Table 1A). In RV, we measured the major (D_maj_) and minor (D_min_) cardiomyocyte dimensions and calculated cardiomyocyte volume, assuming a cell shape in the form of prolate ellipsoid (Supplementary Figures 3B,C) as discussed in Methods (26, 27). The histological measurements showed that the cardiomyocytes volume was significantly higher in RV (*p* ≤ 0.001 males: and *p* ≤ 0.01 females) of coinfected mice compared to uninfected mice (Supplementary Tables 1A,B). We also measured interstitial fibrosis and extracellular space expansion in RV using the microscopic images of Trichrome sections (data not shown) (Supplementary Figure 3). The collagen layer in the extracellular space was significantly expanded (*p* ≤ 0.05) in coinfected males compared to coinfected females. These data suggest that cardiomyocytes size significantly increased in male coinfected mice compared to female coinfected mice, however, the extracellular membrane space significantly disintegrated in between the cardiomyocytes in coinfected female mice compared to coinfected male mice. These data further indicate that coinfected male mice display a hypertrophied cardiomyocyte phenotype and the female coinfected mice display an atrophied cardiomyocyte phenotype. The male coinfected mice showed significantly elevated fibrosis compared to female coinfected mice (Figure 3B). The levels of accumulated lipid droplets in the capillaries, infiltrated immune cells and fibrosis in RV in male coinfected mice were significantly greater compared to female coinfected mice (Figure 3C and Supplementary Figure 2).

The changes in cardiomyocyte size affect the size of the hearts (Supplementary Figure 3). Therefore, we performed the morphometric analysis of the hearts as described in Materials and Methods. The thickness of the left ventricular wall (LVW), right ventricular wall (RVW) and septal wall (SW) differed between males and females and infected and coinfected mice compared to sex matched control mice (Supplementary Table 1B). LVW thickness significantly decreased in female mice singly infected with CoV2 or *T. cruzi* compared to female control mice; however, no significant difference was observed in female coinfected mice. LVW thickness in male CoV2/*T. cruzi* singly infected and coinfected mice showed no significant differences compared to male control mice. RVW thickness significantly decreased in female control mice compared to male control mice, and it was further decreased in female coinfected mice. Interestingly, the thickness of RVW was significantly reduced in male coinfected mice compared to male *T. cruzi* infected mice, which was not observed for female coinfected and *T. cruzi* infected mice. SW thickness increased in female CoV2 mice and was inversely proportional to the decreased LVW thickness compared to female control mice.

### CoV2 Infection Alters Cardiac Lipid Metabolism Differently in Male and Female huACE2 Mice With and Without Indeterminate *T. cruzi* Infection

**Figure 4:**
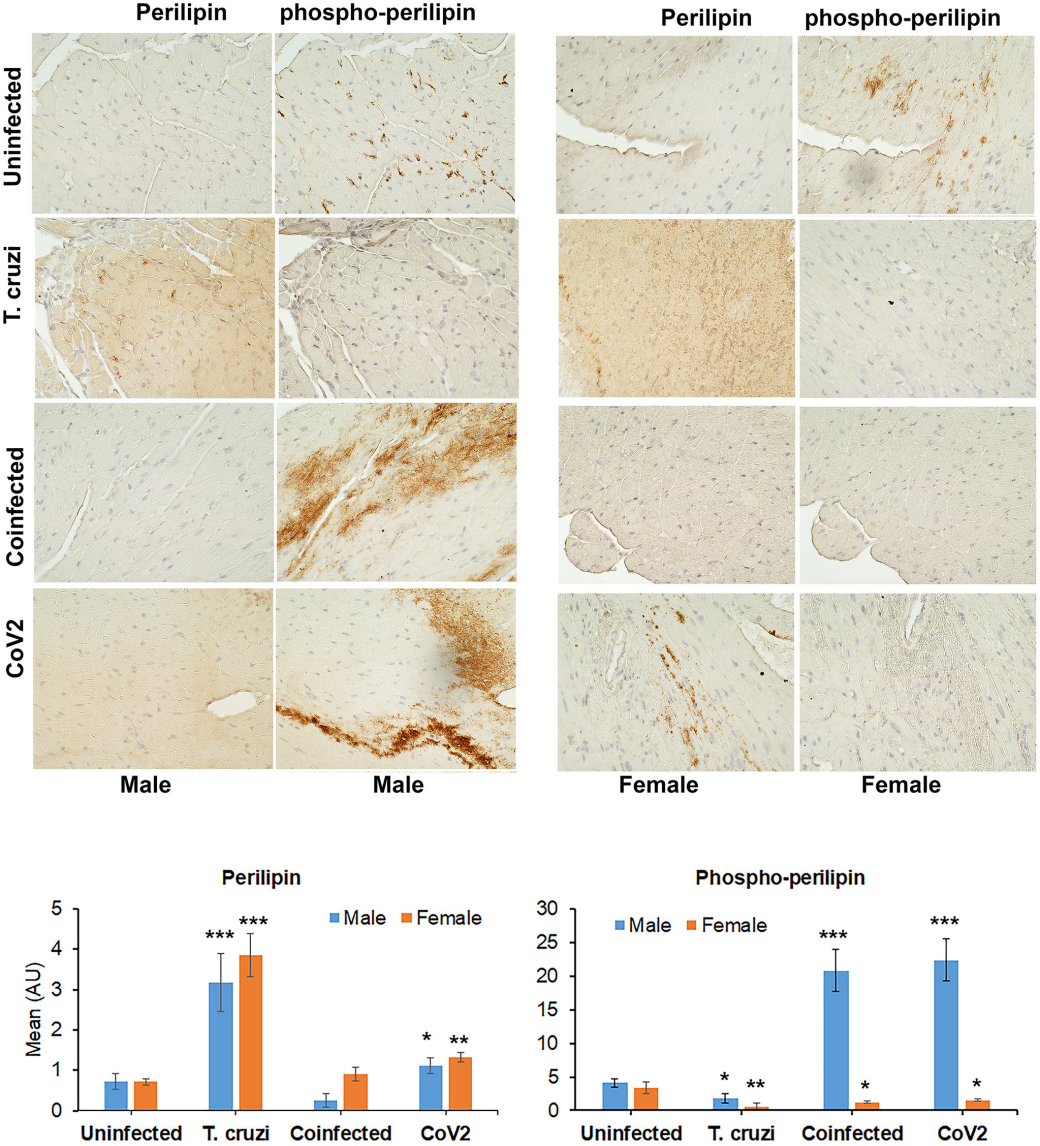
Immunohistochemistry staining (top panel) of huACE2 mouse myocardium using anti-perilipin (left) and anti-phospho-perilipin (right) antibody. The stained sections show altered levels of perilipin and phospho-perilipin during indeterminate *T. cruzi* infection and coinfection in both male and female mice compared to uninfected and CoV2 infected mice. IHC staining intensity (bottom panel) was analyzed using ImageJ software. Positive staining intensities of perilipin and phospho-perilipin were calculated and plotted separately (*n* = 4 mice, a minimum of five images/section were analyzed). The error bars represent standard error of the mean. *p < 0.05, **p < 0.01 and ***p < 0.001 compared with uninfected sex matched mice (M, male; F, female).

Earlier we demonstrated that *T. cruzi* infection causes increased cardiac lipid accumulation, which elevates cardiac mitochondrial and endoplasmic dysfunction (33, 34). We performed immunohistochemistry (IHC) staining of perilipin (lipid droplet associated protein) and phospho-perilipin (marker of break-down of lipid droplets) to analyze the levels of micro-lipid droplets in the myocardium (Figure 4). The IHC analysis showed significantly increased (*p* ≤ 0.001) levels of perilipin and significantly decreased (*p* ≤ 0.001) levels of phospho-perilipin in the myocardium of *T. cruzi* infected mice compared to uninfected mice. However, in the myocardium of coinfected mice, the levels of perilipin significantly decreased (*p* ≤ 0.001) compared to *T. cruzi* infected mice, suggesting that accumulated lipid droplets in the cardiomyocytes were used up during the CoV2 infection (Figure 4). The levels of perilipin also increased in the myocardium of CoV2 infected mice compared to uninfected mice (Figure 4). Interestingly, male CoV2 mice showed significantly greater (*p* ≤ 0.01) levels of phospho-perilipin compared to female CoV2 mice, which is similar to the myocardium of coinfected mice (Figure 4). These data suggest that the myocardium uses accumulated lipids rapidly during CoV2 infection; however, the rate of lipid catabolism may differ between males and females.

### CoV2 Infection Alters Cardiac Adiponectin (C-ApN) Levels and Adiponectin (ApN) Signaling in the Hearts in Coinfected Mice

**Figure 5:**
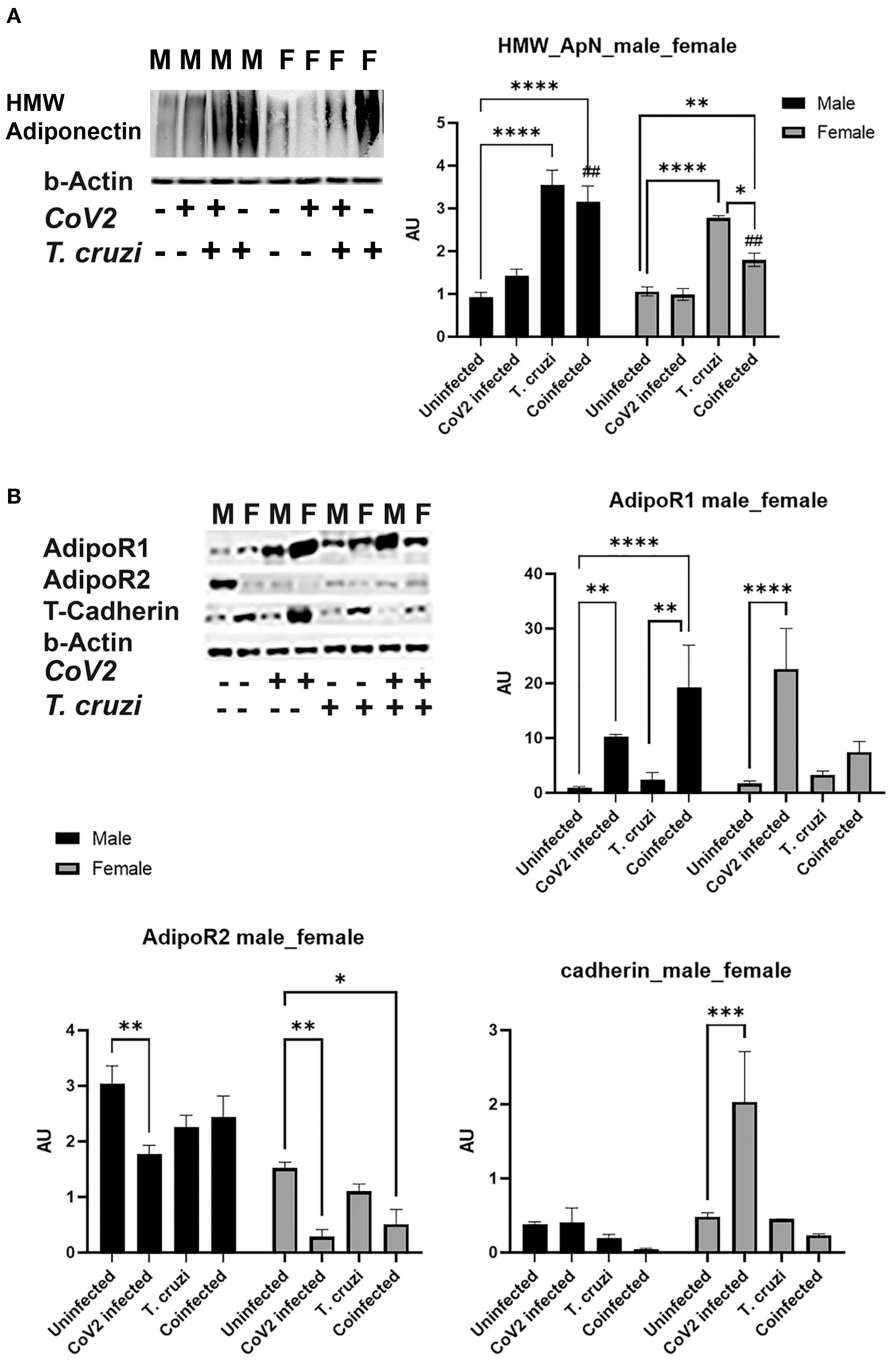
Immunoblot analysis of adipogenic markers in the hearts of Cov2 infected, *T. cruzi* infected, and coinfected huACE2 mice. Western blot images of **(A)** cardiac high-molecular weight adiponectin (HMW ApN) and **(B)** ApN receptors (Adipo R1, R2 and T-cadherin). β-Actin was used as loading control. Fold-changes in the protein levels of adipogenic markers normalized to β-Actin are shown as bar graphs. (A dot plot displaying individual data point is shown in Supplementary Figure 4). The error bars represent standard error of the mean. *p < 0.05, **p < 0.01, ***p < 0.001 and ****p < 0.0001 compared to uninfected sex matched mice (*n* = 4/sex/group) (^*##*^*p* ≤ 0.01 for comparisons between CoV2 infected and coinfected mice) (M, male; F, female).

We detected no change in parasite load in the heart between *T. cruzi* and coinfected mice (data not shown); however, we observed significant heart morphological changes, including accumulation of lipid droplets (Figures 3, 4). Because adiponectin and it's signaling are associated with adipogenesis and lipid oxidation, we examined and quantified the levels of adipogenic markers such as adiponectin (ApN) and its receptors in the hearts by Western blotting in coinfected and CoV2 infected mice and compared with *T. cruzi* infected and uninfected mice (Figure 5). We measured the levels of cardiac high-molecular weight ApN (C-HMW ApN), a.k.a. its anti-inflammatory/anti-fibrotic/metabolically active form (35, 36) by native gel (Figure 5A). Previously we showed a strong correlation between C-ApN levels and progression of cardiomyopathy during CD, wherein elevated levels of C-ApN were associated with mortality due to cardiac dilation (22). As expected, the levels of C-HMW ApN significantly (*p* ≤ 0.0001) increased in the hearts of *T. cruzi* infected mice both in males and females compared to sex matched uninfected mice. However, although the levels of C-HMW ApN significantly increased (*p* ≤ 0.0001) in coinfected male mice compared to uninfected male mice, no significant difference was observed compared to *T. cruzi* infected male mice (Figure 5A). In female coinfected mice, the levels of C-HMW ApN significantly decreased (*p* ≤ 0.05) compared to *T. cruzi* infected female mice; however, these levels were still significantly increased (*p* ≤ 0.01) compared to uninfected female mice (Figure 5A). CoV2 infection did not alter C-HMW-ApN levels in either male or female mice. These data indicate that the levels of anti-inflammatory C-HMW-ApN are high in the hearts of *T. cruzi* infected and coinfected mice and significantly elevated in male mice compared to female mice. The regulatory actions of ApN are mainly mediated by its receptors Adiponectin-R1 and -R2 (Adipo R1 and R2) and T-cadherin (37–39). We found that the levels of AdipoR1, AdipoR2 and T-cadherin were significantly altered in the hearts between the sexes and infections (Figure 5B). In particular, the levels of R1 significantly increased in CoV2 (*p* ≤ 0.01) and coinfected (*p* ≤ 0.0001) male mice compared to their sex matched controls (Figure 5B). In females, AdipoR1 significantly increased (*p* ≤ 0.0001) only in the hearts of CoV2 infected (not in coinfected female mice) compared to uninfected female mice (Figure 5B). Between males and females AdipoR1 significantly (*p* ≤ 0.05) increased in female uninfected and CoV2 infected female mice compared to their male counterparts; however, it was significantly reduced (*p* ≤ 0.05) in coinfected female mice compared to coinfected male mice. The levels of AdipoR2 significantly decreased (*p* ≤ 0.01) in CoV2 infected male and female mice compared to their sex matched uninfected mice (Figure 5B). The levels of AdipoR2 also significantly decreased (*p* ≤ 0.05) in female coinfected mice compared to female uninfected mice. Interestingly, the levels of AdopoR2 were significantly reduced in female mice (uninfected and infected (*T. cruzi*/CoV2/coinfected) compared to their respective male mice (Figure 5B). Another ApN receptor, T-cadherin, significantly increased (*p* ≤ 0.001) only in female CoV2 infected mice compared to uninfected mice. Taken together, these data suggest that C-HMW ApN may regulate anti-inflammatory and metabolic signaling in the hearts of coinfected male mice via AdipoR1.

### Cardiac Immune Signaling Differs Between Male and Female Coinfected and CoV2 Infected Mice

**Figure 6:**
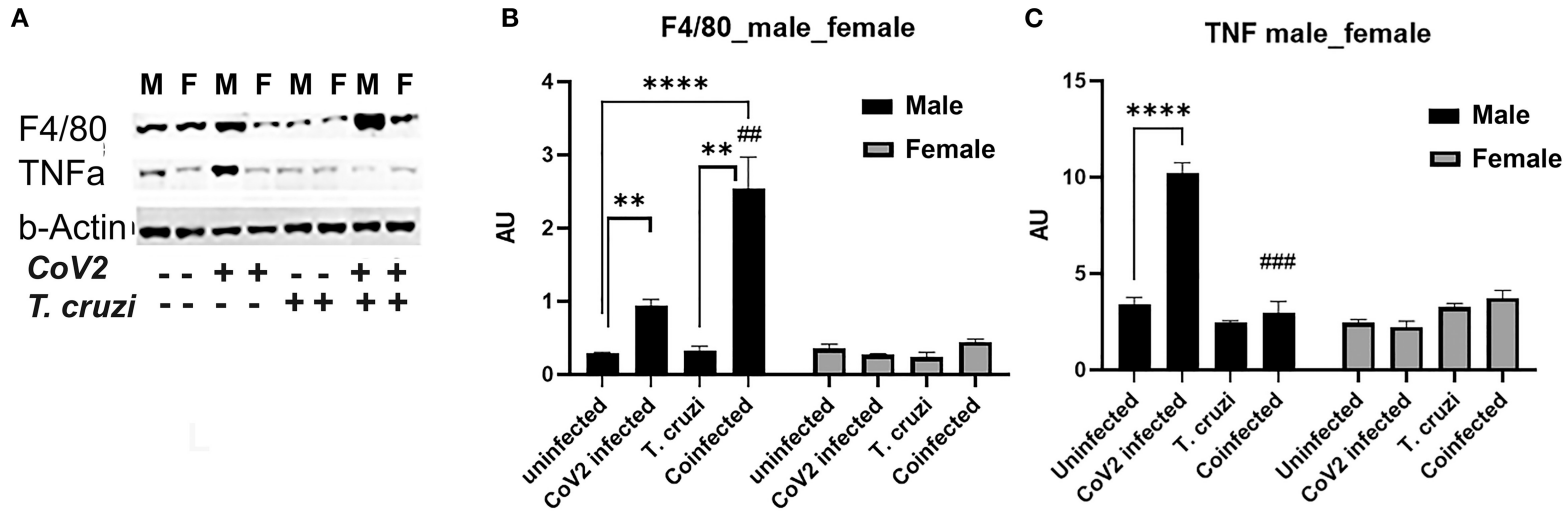
Immunoblot analysis of inflammatory markers in the hearts of CoV2 infected, *T. cruzi* infected, and coinfected huACE2 mice. Western blot images **(A)** of macrophage marker (F4/80) and proinflammatory cytokine TNFα. β-Actin was used as loading control. Fold changes in the protein levels of F4/80 **(B)** and TNFα **(C)** were normalized to β-Actin expression and are shown as bar graphs (A dot plot displaying individual data point presented in Supplementary Figure 4). The error bars represent standard error of the mean. **p < 0.01 and ****p < 0.0001 compared to uninfected sex matched mice (*n* = 4/sex/group). (^*##*^*p* ≤ 0.01, and ^*###*^*p* ≤ 0.001 for comparisons between CoV2 infected and coinfected mice) (M, male; F, female).

Because we observed significant changes in the levels of HMW adiponectin in the hearts during infection, which may affect immune signaling, we analyzed the levels of infiltrated macrophages and the levels of proinflammatory TNFα in the hearts by immunoblot analysis (Figure 6A). The cardiac levels of F4/80 significantly increased in CoV2 infected (*p* ≤ 0.01) and coinfected (*p* ≤ 0.0001) male mice compared to sex matched uninfected mice. The levels of F4/80 significantly increased (*p* ≤ 0.01) in the hearts of coinfected male mice compared to CoV2 singly infected male mice (Figures 6A,B), suggesting significant infiltration of macrophages in the hearts of coinfected male mice (but not in coinfected female mice). The levels of F4/80 significantly increased (*p* ≤ 0.05) in singly CoV2-infected and coinfected males compared to singly CoV2-infected and coinfected females (Figures 6A,B). Interestingly, the cardiac levels of TNFα significantly increased *(p* ≤ 0.0001) in CoV2 infected male mice but not in CoV2 infected female mice compared to their respective sex matched uninfected mice (Figures 6A,C). The cardiac levels of TNFα significantly decreased (*p* ≤ 0.001) in coinfected male mice compared to CoV2 infected male mice (Figures 6A,C). These data suggested that although the levels of infiltrated immune cells are increased in the hearts of male coinfected mice during CoV2 infection, the levels of pro-inflammatory TNF are decreased, which may be due to the increased levels of cardiac HMW adiponectin that can regulate macrophage activation (36, 40).

### CoV2 Infection Differently Alters Cardiac Lipid and Glucose Metabolism in the Hearts of Coinfected Male and Female Mice

**Figure 7:**
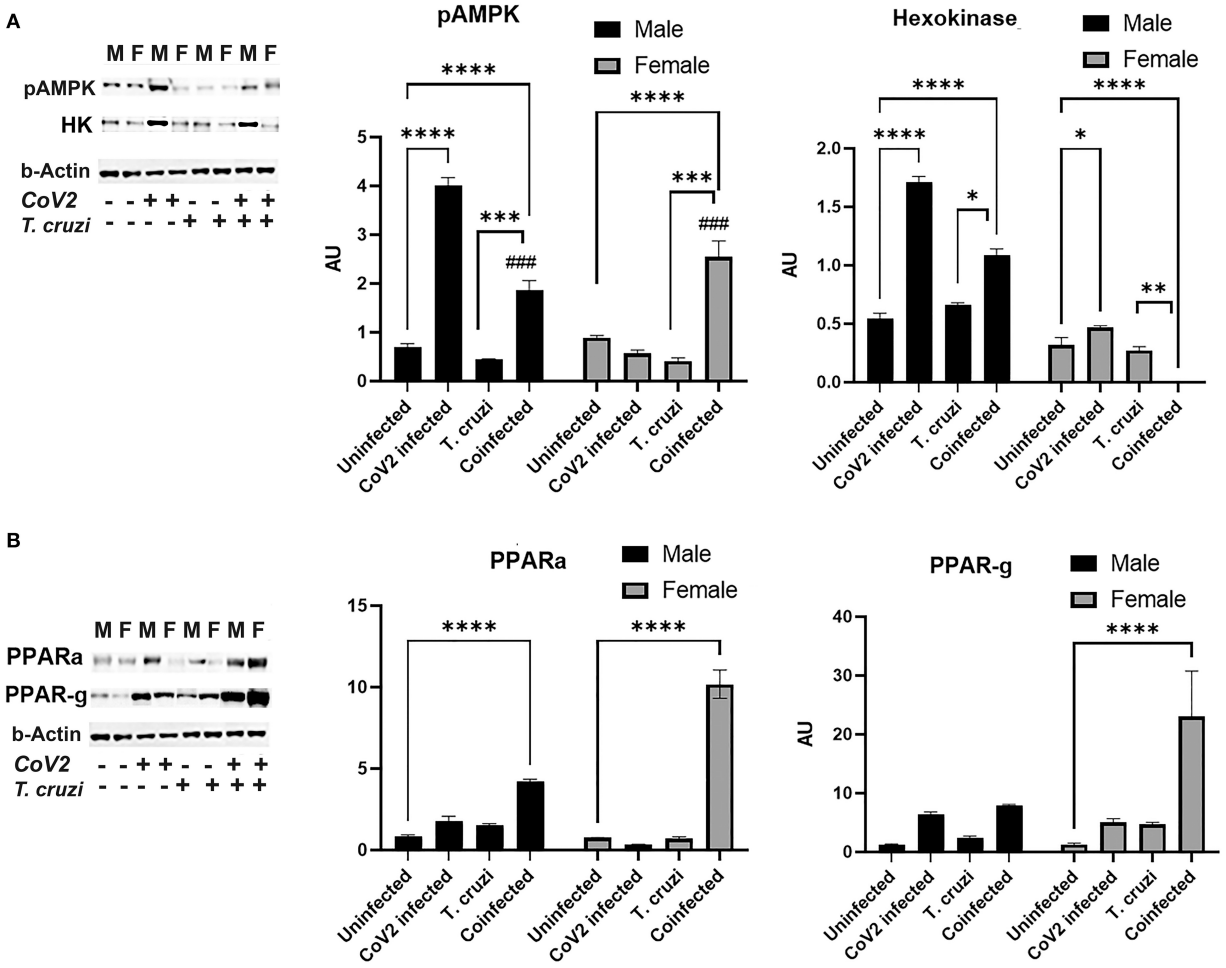
Immunoblot analysis of markers of glucose and lipid metabolism in the hearts of CoV2 infected, *T. cruzi* infected, and coinfected huACE2 mice. Immunoblot images of **(A)** markers of glucose metabolism (pAMPK and hexokinase II) and **(B)** markers of lipid metabolism (PPARα and PPARγ). β-Actin was used as loading control. Fold changes in the protein levels normalized to β-Actin are shown as bar graphs (A dot plot displaying individual data points is presented in Supplementary Figure 4). The error bars represent standard error of the mean. *p < 0.05, **p < 0.01, ***p < 0.001 and ****p < 0.0001 compared to uninfected sex matched mice (*n* = 4/sex/group) (^*###*^*p* ≤ 0.001 for comparisons between CoV2 infected and coinfected mice) (M, male; F, female).

ApN regulates glucose (AMPK/glycolysis) and lipid (PPARs) metabolism (41–44). Therefore, we analyzed heart levels of pAMPK and hexokinase II (HK) as markers of glucose metabolism and PPARα and PPARγ as markers of lipid oxidation and lipogenesis, respectively (Figure 7). Western blotting analysis demonstrated significantly increased pAMPK (*p* ≤ 0.0001) in the hearts of coinfected male and female mice compared to their respective sex matched uninfected controls (Figure 7A). Another marker of glycolysis, hexokinase-2 (HK2), significantly increased in coinfected male mice (*p* ≤ 0.001) and significantly decreased in coinfected female mice compared to sex matched uninfected mice (Figure 7A). These data suggest that glycolysis is significantly increased in coinfected male mice and significantly decreased in coinfected female mice. Infection with CoV2 alone significantly increased pAMPK (*p* ≤ 0.0001) only in male mice (compared to uninfected male mice), and its levels were significantly reduced (*p* ≤ 0.0001) in CoV2 infected female mice compared to CoV2 infected male mice (Figure 7A). The levels of HK also significantly decreased (*p* ≤ 0.0001) in CoV2 infected female mice compared to CoV2 infected male mice. These data suggest that, in general during CoV2 infections, the myocardium of male mice shifts their energy resources toward glycolysis, whereas the myocardium of female mice shuts down the glycolysis pathway.

ApN/AMPK signaling also regulates lipid metabolism. Western blotting analysis demonstrated significantly increased PPARα in the hearts of both coinfected male (*p* ≤ 0.0001) and coinfected female (*p* ≤ 0.0001) mice compared to sex matched uninfected mice. However, the levels of PPARα in the hearts of coinfected female mice was significantly higher (*p* ≤ 0.0001) compared to coinfected male mice. CoV2 infection alone also significantly increased (*p* ≤ 0.05) cardiac PPARα levels in male mice compared to uninfected male mice (Figure 7B). The cardiac levels of PPARγ significantly increased (*p* ≤ 0.0001) only in coinfected female mice compared to uninfected female mice and other groups (Figure 7B). These data suggest that both lipogenesis and lipid oxidation dominate in the hearts of coinfected female mice, and that these hearts mainly depend on lipid oxidation and utilization as their energy resource during CoV2 infection, whereas coinfected male mice hearts may utilize both glucose and lipids as sources of energy.

### Beta-Adrenergic Receptors Play a Major Role in the Hearts of Coinfected Female Mice

**Figure 8:**
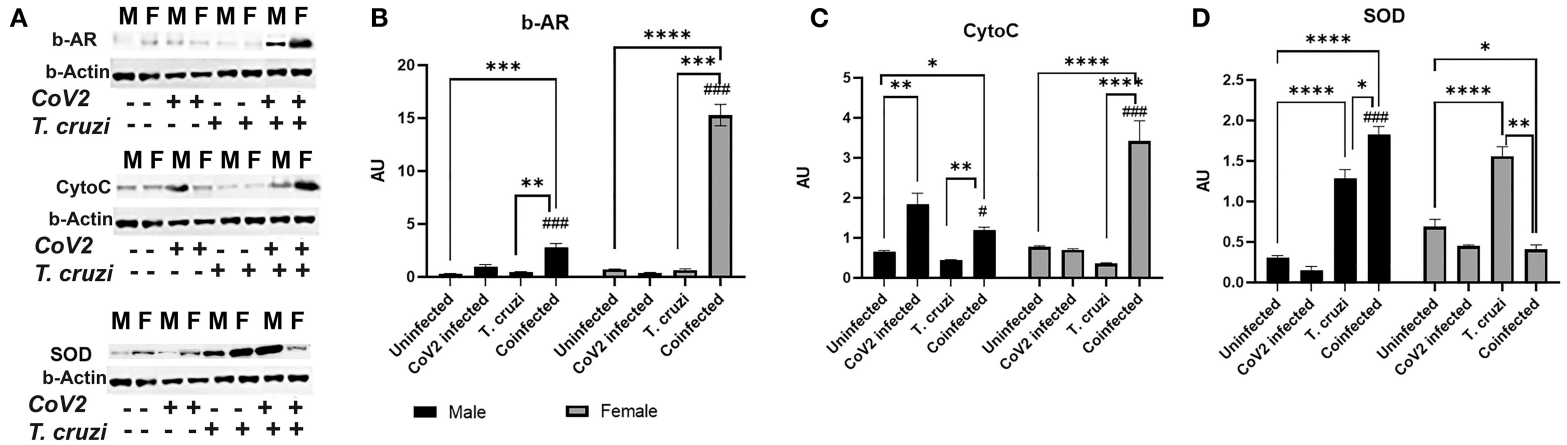
Immunoblot analysis of β-Adrenergic Receptor and markers of mitochondrial β-oxidation in the hearts of CoV2 infected, *T. cruzi* infected, and coinfected huACE2 mice. Western blot images **(A)** of β-Adrenergic Receptor (β-AR), cytochrome C (Cyto C), and superoxide dismutase (SOD). β-Actin was used as loading control. Fold changes in the protein levels of β-AR **(B)**, Cyto C **(C)** and SOD **(D)** were normalized to β-Actin expression and are shown as bar graphs (A dot plot displaying individual data points is presented in Supplementary Figure 4). The error bars represent standard error of the mean. *p < 0.05, **p < 0.01, ***p < 0.001 and ****p < 0.0001 compared to uninfected sex matched mice (*n* = 4/sex/group) (^#^*p* ≤ 0.05 and ^*###*^*p* ≤ 0.001 for comparisons between CoV2 infected and coinfected mice) (M, male; F, female).

Beta-adrenergic receptors (b-AR) are implicated in various heart diseases (45, 46), and several studies have demonstrated that chronic *T. cruzi* infection causes dysfunctional b-AR signaling in the hearts in Chagas' mouse models (47–49). Increased b-AR activity causes lipolysis resulting in the release of free fatty acids and their derivatives, which are the ligands for PPARα and PPARγ (50–52). PPARα is involved in fatty acid oxidation, whereas PPARγ is implicated in lipogenesis (53–56). Our data also indicated that increased PPARα and PPARγ expression in the hearts of female coinfected mice may be induced via a different mechanism and not via the HMW-ApN-AMPK axis (Figure 7). Because b-AR are highly sensitive to estrogen (57–59), we investigated whether b-AR play an important role in inducing PPAR levels in female mice by Western blotting analysis (Figure 8). We observed significantly increased (*p* ≤ 0.05) b-AR in female uninfected mice compared to male uninfected mice, indicating that females may be more sensitive to b-AR stimulation. The levels of b-AR significantly increased in the hearts of coinfected mice both in males (*p* ≤ 0.0001) and females (*p* ≤ 0.001) compared to their sex matched uninfected mice (Figure 8A). However, the levels of b-AR significantly increased (*p* ≤ 0.0001) in the hearts of coinfected female mice compared to coinfected male mice, suggesting that in female coinfected mice b-AR activation may significantly increase cardiac lipolysis and lipid metabolism (Figures 7, 8A).

The cardiac levels of PPARγ and PPARα significantly increased (*p* ≤ 0.0001) in coinfected female mice compared to coinfected male mice and other groups (Figure 7). Because increased PPARs may elevate lipid mitochondrial β-oxidation, we analyzed the levels of Cytochrome C (Cyto) and superoxide dismutase (SOD) (Figure 8A). Western blotting analysis showed a significant increase in Cyto (*p* ≤ 0.05) and SOD (*p* ≤ 0.0001) in the hearts of coinfected male mice and a significant increase in Cyto (*p* ≤ 0.0001) but a significant decrease in SOD (*p* ≤ 0.05) in the hearts of coinfected female mice compared to their respective sex matched uninfected mice (Figures 8B,C). In fact, the levels of Cyto significantly increased (*p* ≤ 0.0001) in the hearts of coinfected female mice compared to coinfected male mice, suggesting greater levels of mitochondrial oxidative phosphorylation in the hearts of coinfected female mice. *T. cruzi* infected mice (both males and females) showed significantly increased SOD (*p* ≤ 0.0001) in the hearts during the indeterminate stage compared to uninfected mice. Together, these data suggested that lipid catabolism and oxidation are higher in female hearts compared to male hearts in coinfected mice, which may prevent the progression of cardiac dilation due to intracellular lipotoxicity (60), but may also cause increased oxidative stress. The reduced levels of SOD in the hearts in coinfected female mice also suggest increased oxidative stress and mitochondrial dysfunction in these mice. Increased mitochondrial dysfunction is associated with cachexia and atrophy (61, 62). On the other hand, in coinfected males, the cardiac accumulation of HMW-ApN and AMPK activation may increase glycolysis and also adipogenesis, which may cause lipotoxicity leading to cardiac steatosis, hypertrophy and early dilated cardiomyopathy in post-COVID mice.

## Discussion

Many clinical and *in vivo* studies have examined the effect of comorbidities, such as diabetes, asthma, hypertension, and cardiac diseases, on the pulmonary pathogenesis and susceptibility to CoV2 infection. However, the effects of metabolic and immunologic changes associated with chronic infectious disease on the risk of developing severe COVID have not been extensively investigated and neither have been the post-COVID effects on the manifestation/activation of other infectious diseases. The present study examines: (i) the effect of *T. cruzi* infection-induced immune and metabolic responses on the susceptibility to CoV2 infection and (ii) the early effect of CoV2 infection on the pathogenesis and risk of developing cardiomyopathy in *T. cruzi* infected mice coinfected with CoV2 during the indeterminate CD stage. We also examined the effects of CoV2 infection on the morphology and metabolic signaling in the hearts of non-CD mice and compared the results with our coinfection model to evaluate the role of *T. cruzi* infection-induced immune and metabolic changes in regulating cardiac viral load and inflammation during CoV2 infection using hACE2 murine models. Moreover, this study assessed whether the relationship between *T. cruzi* and CoV2 infections differs between male and female mice. Specifically, to understand the interplay between *T. cruzi* and CoV2 infections, we used transgenic hACE2 mice (males and females) nasally infected with SARS-CoV2 in mice pre-infected with *T. cruzi*. Our study revealed that: (a) *T. cruzi*-CoV2 coinfection increased ACE2 levels in the lungs and hearts, but the viral load significantly decreased compared to CoV2 infection alone, and (b) there was no observed difference between viral loads in the hearts of male and female coinfected/CoV2 mice. However, CoV2 infection differently altered immune and metabolic status in the hearts of male and female mice (both in CoV2 and coinfection models). More importantly, our study showed that the impact of CoV2 infection on cardiac metabolism and progression of dilated CCM in coinfected mice is sex-dependent: male coinfected mice were more susceptible to developing hypertrophied dilated CCM, whereas female coinfected mice were more susceptible to developing cardiac cachexia.

The viral load in the lungs in female CoV2 infected mice were significantly lower compared to male CoV2 infected mice, which is reminiscent to the observations made in COVID patients (19). Some clinical studies also suggested that the mortality rate of women in young COVD-19 patients is lower than that of men, while there is no difference between the mortality rate of women and men in elderly patients (63, 64). However, not many clinical studies suggesting sex difference in cardiac pathology during COVID are reported. Our study shows for the first time that the viral load in the hearts of coinfected mice is lower compared to CoV2 singly infected mice, although the cardiac levels of ACE2 were similar (in males) or greater (in females) in coinfected mice compared to CoV2 singly infected mice. The significantly reduced viral load in the hearts of coinfected mice may be due to the altered immune and metabolic changes caused by *T. cruzi* infection during acute and indeterminate stages. Similar to the hearts, we also observed significantly reduced viral loads in the lungs of coinfected male mice compared to CoV2 singly infected mice, even though the levels of ACE2 were significantly greater in coinfected male mice. These data suggest that the rates of viral entry, infection, severity, and pathology are not regulated just by the levels of ACE2 receptor, and that the immune and metabolic status of the organ may play a major role in regulating COVID pathology.

*T. cruzi* infection causes increased accumulation of lipid droplets in the capillaries and micro-lipid droplets in the cardiomyocytes in the myocardium, which in turn alters cardiac metabolic status (Figures 2, 4) (22, 33). The increase in lipid accumulation in the myocardium in *T. cruzi* infected mice may be due to the increased cardiac HMW-ApN levels, which regulates adipogenesis, lipid oxidation, and anti-inflammatory signaling (as shown by increased SOD and reduced TNFα levels) during the indeterminate stage (22, 34). The levels of HMW-ApN in the hearts of coinfected mice were significantly greater compared to CoV2 alone infected mice, which might have contributed to the reduced TNFα levels (although there was increased infiltration of macrophages) in the hearts of coinfected mice (Figures 3, 5, 6). Interestingly, although we observed a similar pattern of altered HMW-ApN and reduced TNFα in the hearts of coinfected males and females, histological and morphological analyses showed significant differences between cardiomyocyte cell size, cell number and presence, localization, and size of lipid droplets between the sexes. The hearts of coinfected male mice showed a hypertrophied and dilated myocardium phenotype, whereas coinfected female mice displayed an atrophied and cachexic myocardium phenotype (Supplementary Figure 3 and Supplementary Table 1A). We also observed a shrunken heart phenotype in coinfected female mice (Supplementary Table 1B). This was reminiscent of cardiac atrophy observed in cancer patients (65).

**Figure 9:**
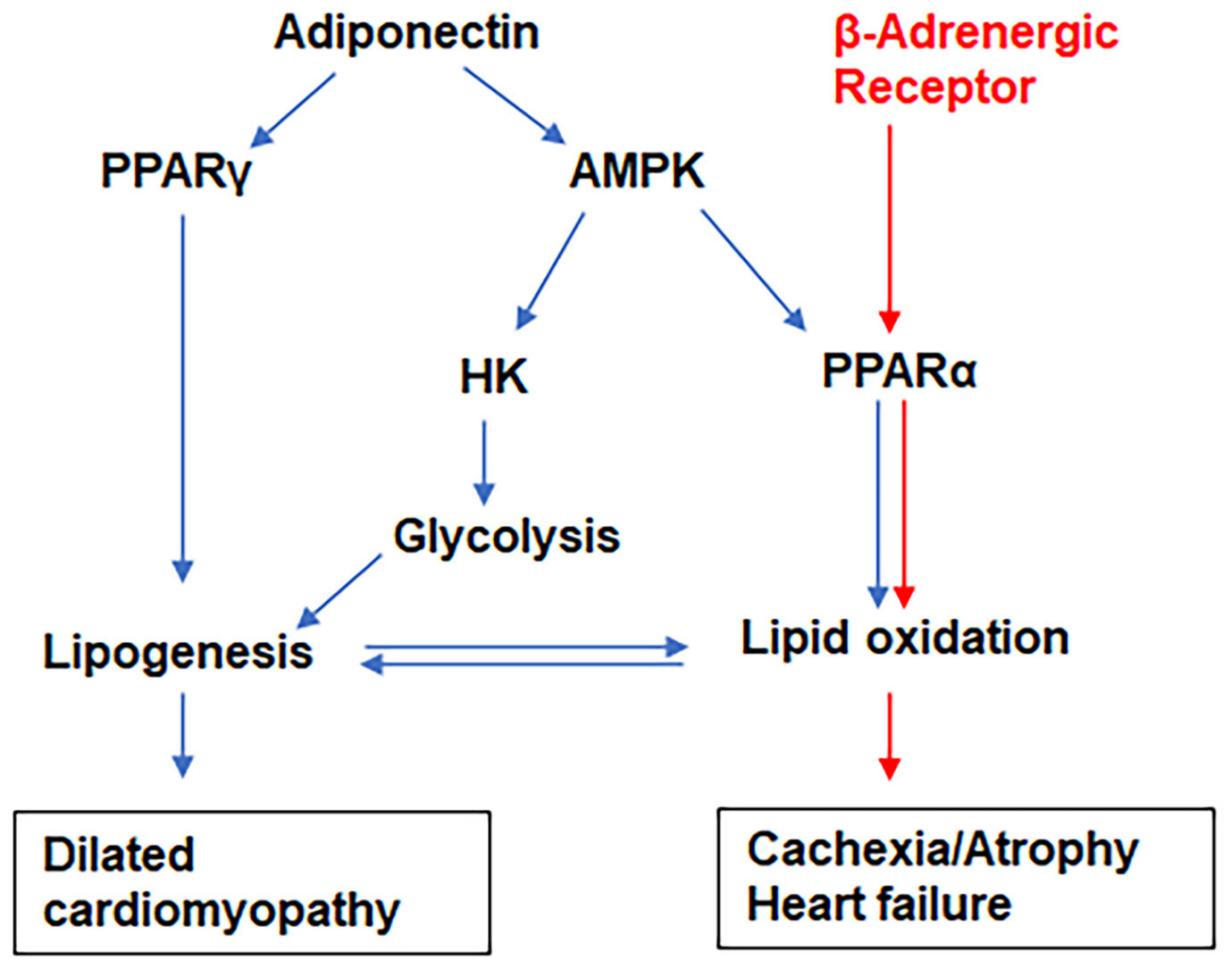
The proposed models of signaling pathways that differentiate the pathogenesis of dilated cardiomyopathy (indicated by blue arrows) and cachexia (indicated by red arrows) in male and female coinfected mice, respectively. The adiponectin/AMPK/glycolysis pathway dominates in the hearts of male coinfected mice, whereas β-adrenergic receptor signaling activates lipid metabolic pathways in female coinfected mice.

CD has been tightly linked to alterations in metabolic substrate utilization and impairments of mitochondrial oxidative capacity and endoplasmic reticulum functions in the hearts (22, 33, 66–68). It is known that patients with heart disease exhibit a shift from fatty acid oxidation toward greater dependance on glucose as a source for cellular energy adenosine triphosphate (ATP) (69). Thus, mitochondria have become a key target in combating this metabolic reprogramming in heart disease including CD (66). Exhaustion of mitochondria or increased mitochondrial oxidative stress results in mutations in mitochondrial DNA, mitochondrial dysfunction and oxidative stress causing cachexia, cardiac atrophy and heart failure (70). During viral and parasitic infections, the myocardium tends to shift its energy utilization from fatty acid oxidation to glycolysis. Increased activation of AMPK in the hearts of infected mice is an indicator of depletion of ATP (23, 71–73). Although the levels of adiponectin and activated AMPK significantly increased in the hearts of both male and female coinfected mice, the downstream mechanism(s) of HMW-ApN/AMPK pathway significantly differed between male and female coinfected mice (Figure 9), in that in male coinfected mice AMPK/PPARα and AMPK/glycolysis increased, whereas in female coinfected mice AMPK/PPARα/PPARγ increased. This may be due to the significant increase in b-AR in coinfected female mice. Thus, in coinfected female mice even in the presence of AMPK activation, glycolysis was completely turned off due to a strong increase in b-AR induced lipolysis (PPARs) and the myocardium may have used only fatty acids as their energy source. In female mice, increased estrogen levels could stimulate b-AR (74), which can activate lipolysis and release free fatty acids, positively regulating PPARs (75, 76). The increased activation of PPARγ and PPARα (in the myocardium in female coinfected mice) may form a vicious cycle in inducing lipogenesis and lipid β-oxidation, respectively, overburdening mitochondrial oxidative phosphorylation and causing dysfunctional mitochondria, cachexia and atrophy. The reduced levels of cardiac SOD (even with increased levels of Cyto), reduced coloration of eosin staining in the heart sections, and decreased cardiomyocyte size (heart size) in the hearts of coinfected female mice suggest oxidative stress, cachexia and atrophy, respectively. Overall, these data suggest that CoV2 infection and coinfection with *T. cruzi* differently affect cardiac metabolic and immune status in male and female mice via host C-ApN/AMPK and b-AR/PPAR-signaling, respectively. Thus, the C-ApN/AMPK and b-AR/PPAR downstream signaling may play major roles in determining the progression, severity, and phenotype of CCM and heart failure in the context of COVID.

The present study investigated the immediate effect of CoV2 infection on heart pathology in CoV2/*T. cruzi* single infections and CoV2 coinfection in the *T. cruzi* indeterminate model, whereas any potential long-term effects remain to be explored. Further studies including a greater number of male and female mice at different time stamps are warranted to evaluate the long-term post-COVID effects on the development and progression of Chagas cardiomyopathy. Our data from this pilot study indicate that the risk of developing dilated cardiomyopathy in *T. cruzi* infected males may be greater than in females and that the risk of developing cachexia-associated heart failure in *T. cruzi*-CoV2 coinfected females may be greater than in coinfected males.

## Data Availability Statement

The original contributions presented in the study are included in the article/Supplementary Materials, further inquiries can be directed to the corresponding author.

## Ethics Statement

The animal study was reviewed and approved by Hackensack University IACUC committee.

## Author Contributions

JN contributed to conception, study design, funding acquisition, supervision, validation, and writing/reviewing the manuscript. DP contributed to the validation and reviewing the manuscript. DD and KL performed the investigation and data analysis. NO performed formal data analysis. HT performed the investigation, data analysis and writing the manuscript. ED provided technical help. All authors contributed to the article and approved the submitted version.

## Funding

This study was supported by grants from the National Institute of Allergy and Infectious Diseases (National Institutes of Health AI150765-01) to JN.

## Conflict of Interest

The authors declare that the research was conducted in the absence of any commercial or financial relationships that could be construed as a potential conflict of interest.

## Publisher's Note

All claims expressed in this article are solely those of the authors and do not necessarily represent those of their affiliated organizations, or those of the publisher, the editors and the reviewers. Any product that may be evaluated in this article, or claim that may be made by its manufacturer, is not guaranteed or endorsed by the publisher.

## Supplementary Material

Supplementary Figure 1 Flow chart of the experimental design.

Supplementary Figure 2 H&E sections (top panel) and Masson trichrome images (bottom panel) of right ventricles (RV) of coinfected male and female mice showing fibrosis (blue and purple), infiltration of immune cells (yellow arrow), accumulation of lipid droplets (red arrow), and enlarged nuclei (green arrowhead) (40X magnified; bar-50μm).

Supplementary Figure 3 Alterations in cardiomyocyte size during CoV2/T. cruzi infection and coinfection in male and female mice: **(A)** H&E sections showing the average cell size in left ventricles (cardiomyocytes - green arrowhead; endothelial cells - yellow arrowhead; macro-lipid droplets - black arrowhead; and immune cells - red arrowhead) (40X magnified; bar-50μm). **(B)** Masson trichrome images (gray scale) of right ventricles showing changes in extracellular collagen layer (white) and thickness of intercellular collagen network during infection and coinfection (40X magnified; bar-50μm). **(C)** Illustration of cardiomyocyte hypertrophy determination by measuring cardiomyocyte length (Dmaj) and diameter (Dmin) and intercellular collagen thickness using histological sections (Masson trichrome) of the hearts. The data are presented as Supplementary Table 1A.

Supplementary Figure 4 Changes in the levels of ACE2 and metabolic signaling markers (Western blot analysis) in the heart/lungs are compared between male and female mice during CoV2/T. cruzi infections and coinfection. Fold changes in the protein levels were normalized to GDI and are represented as a dot plot. The error bars represent standard error of the mean. ^*^*P* < 0.05, ^**^*P* < 0.01, ^***^*P* < 0.001 and ^****^*P* < 0.0001compared between males and females in each group.

Supplementary Table 1A Cardiomyocyte volume by histology. Cardiomyocyte length and width were measured (*n* = 40 cells/sex/group) on microscopic images of the cardiac histology sections (right ventricles) and the volume was calculated as described in Methods. The significance *p*-value for differences in volume was calculated by *t*-test comparing each group to sex matched uninfected mice and is denoted by “^*^” (^*^*p* ≤ 0.05, ^**^*p* ≤ 0.01 and ^***^*p* ≤ 0.001).

Supplementary Table 1B Morphometric analysis of the hearts of CoV2/T. cruzi infected and coinfected male and female mice. The thickness of the right ventricle wall (RVW), left ventricle wall (LVW) and intra-septal wall (Septal -W) was measured as mentioned in materials and methods and is presented in mm. The significance p-value for differences in wall thickness was calculated by t-test comparing each group to sex matched uninfected mice and is denoted by “^*^” (^*^*p* ≤ 0.05, ^**^ *p* ≤ 0.01 and ^***^*p* ≤ 0.001). The significance p-value for differences in wall thickness between coinfected and sex matched T. cruzi infected mice is denoted by “#” (# *p* ≤ 0.05).
