# Supplementary material for: Sex Differences in Cardiac Pathology of SARS-CoV2 Infected and Trypanosoma cruzi Co-infected Mice
Source: Front Cardiovasc Med. 2022 Mar 11;9:783974. doi: 10.3389/fcvm.2022.783974 (PMC8965705; doi:10.3389/fcvm.2022.783974)
Supplement: Supplementary Figure 1 — Flow chart of the experimental design. [file Data_Sheet_1.PDF]

Supplemental Fig. 1:
Experimental design

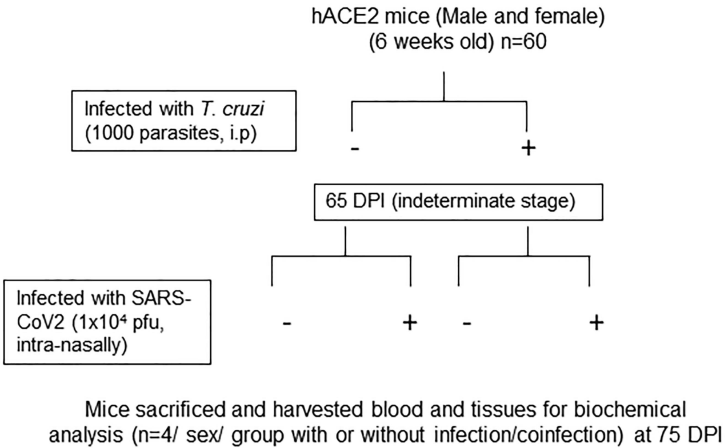

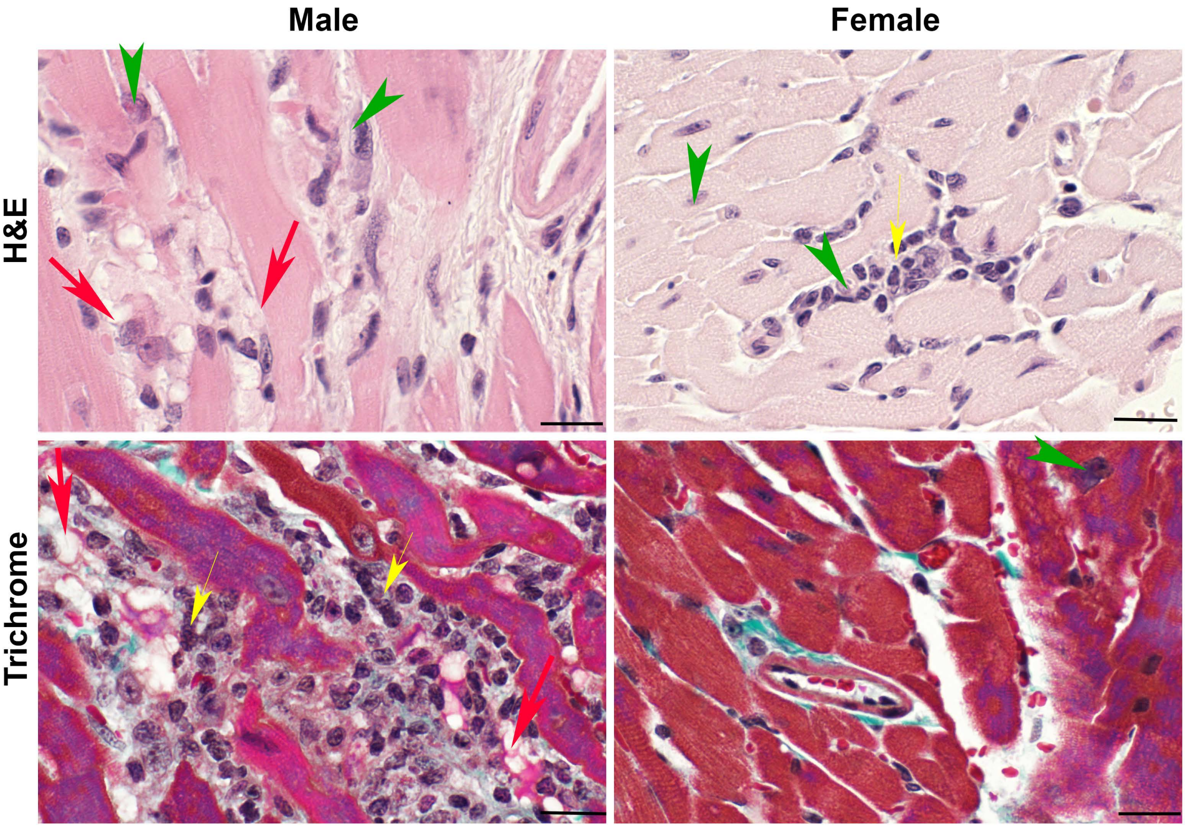
Male
Female
H&E
Trichrome

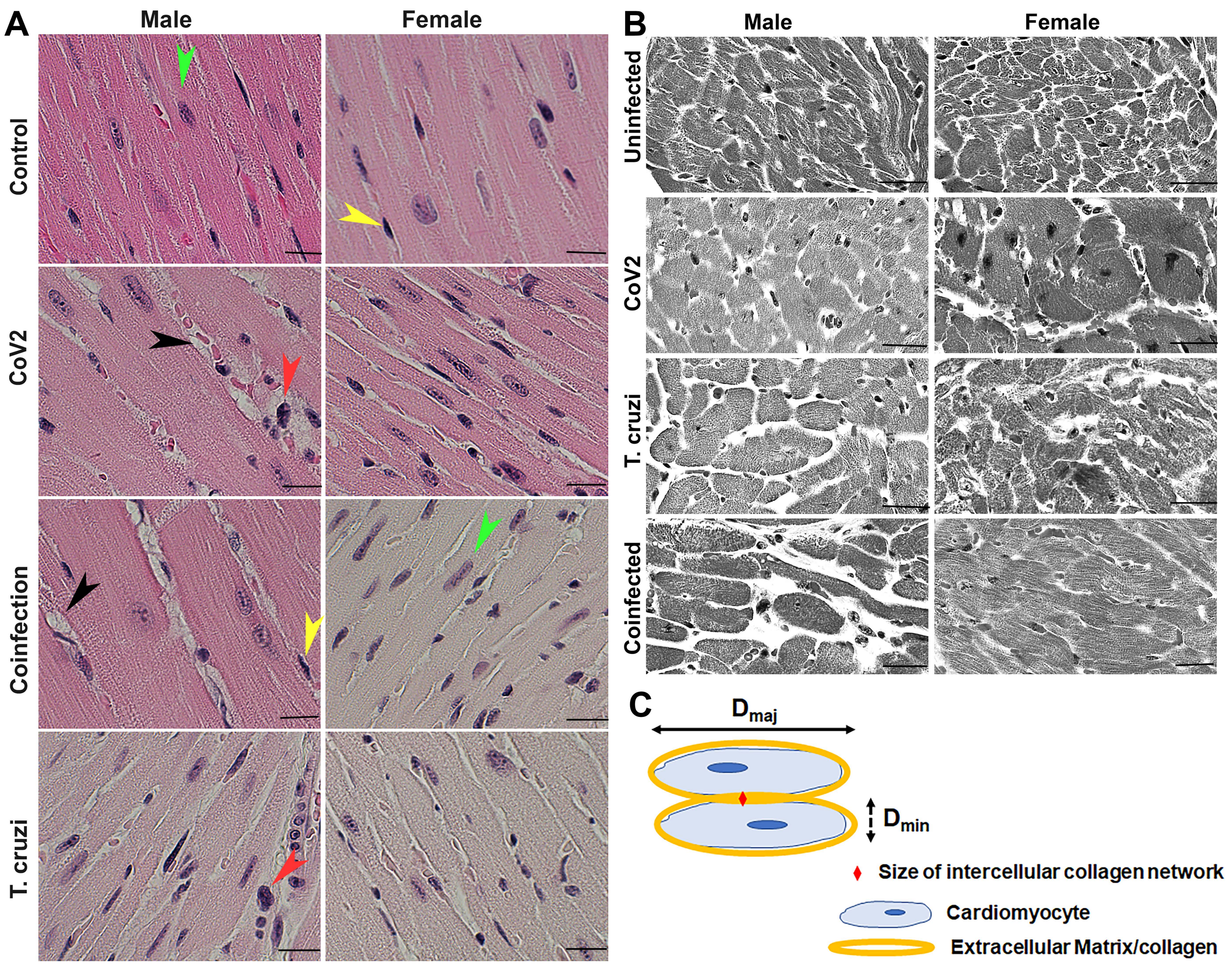
A
Male
Female
Control
CoV2
Coinfection
T. cruzi
B
Male
Female
Uninfected
CoV2
T. cruzi
Coinfected
C
Dmaj
Dmin
Size of intercellular collagen network
Cardiomyocyte
Extracellular Matrix/collagen

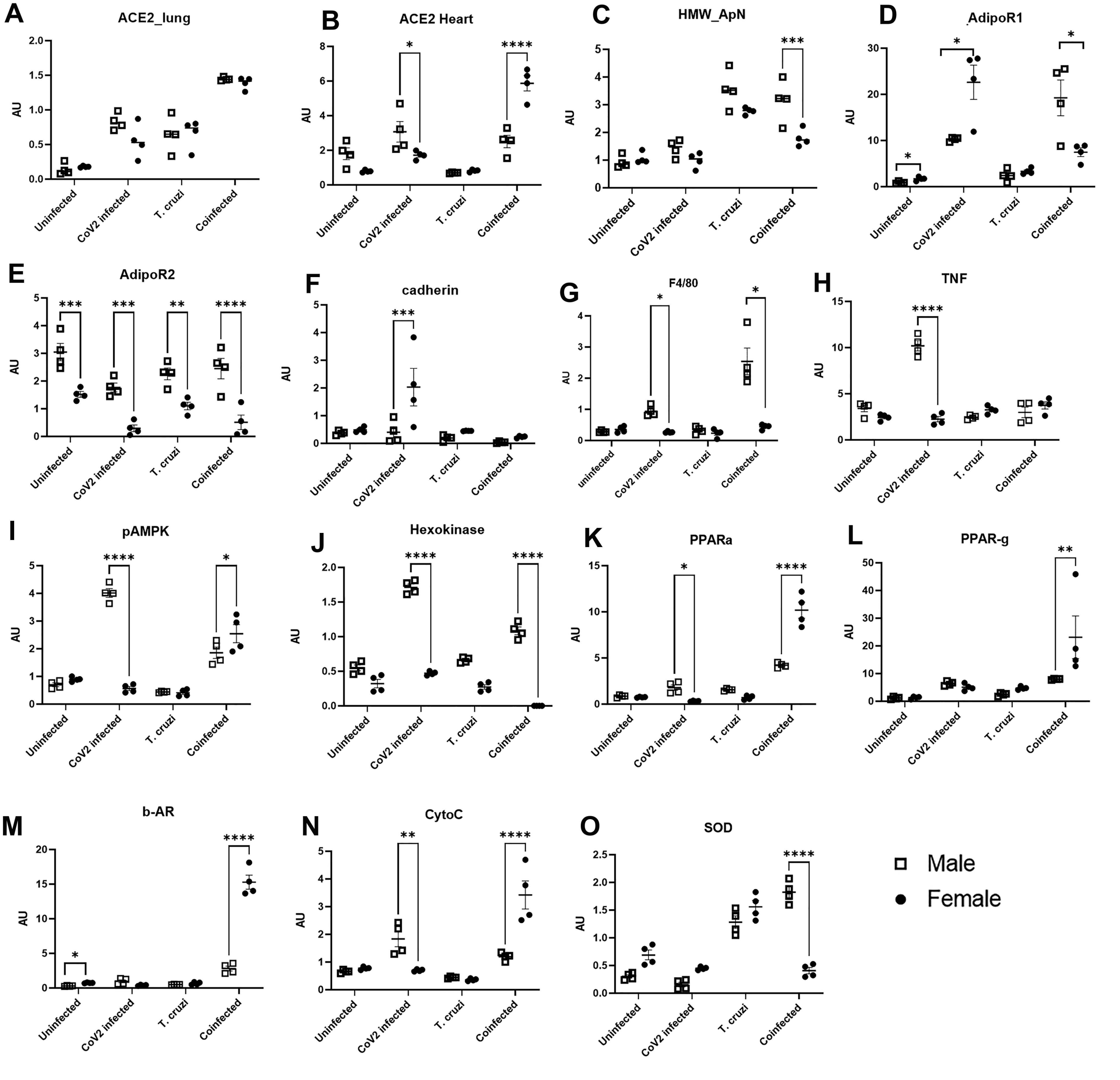

A
ACE2_lung
B
ACE2 Heart
C
HMW_ApN
D
AdipoR1
E
AdipoR2
F
cadherin
G
F4/80
H
TNF
I
pAMPK
J
Hexokinase
K
PPARa
L
PPAR-g
M
b-AR
N
CytoC
O
SOD
AU
Uninfected
CoV2 infected
T. cruzi
Coinfected
Male
Female
