# Supplementary material for: Sex Differences in Cardiac Pathology of SARS-CoV2 Infected and Trypanosoma cruzi Co-infected Mice
Source: Front Cardiovasc Med. 2022 Mar 11;9:783974. doi: 10.3389/fcvm.2022.783974 (PMC8965705; doi:10.3389/fcvm.2022.783974)
Supplement: Supplementary Table 1A — Cardiomyocyte volume by histology. Cardiomyocyte length and width were measured (n = 40 cells/sex/group) on microscopic images of the cardiac histology sections (right ventricles) and the volume was calculated as described in Methods. The significance p-value for differences in volume was calculated by t-test comparing each group to sex matched uninfected mice and is denoted by “*” (*p ≤ 0.05, **p ≤ 0.01 and ***p ≤ 0.001). [file Table_1.doc]

**Table. 1A: Cardiomyocyte volume by histology**

| **Mice** | **Male**  **(x10^4^ um^2^)** | **Female**  **(x10^4^ um^2^)** |
| --- | --- | --- |
| **Uninfected**  **CoV2 infected**  **T. *cruzi* infected**  **Coinfected** | 6.4±1.2  13.3±2.3**  9.8±2.6  17.17±2.8*** | 4.37±1.8  10.2±2.2*  5.66±2.0  7.29±1.4** |

* p<0.05, ** p<0.01, and *** p<0.001 compared to uninfected sex matched mice (n=4/sex/group).

**Table. 1B: Morphometric analysis of the hearts of CoV2/*T. cruzi* infected and coinfected male and female mice.**

|  | **Male Control** | **Female Control** | **Male CoV2** | **Female CoV2** | **Male T.cruzi** | **Female T.cruzi** | **Male Coinfect** | **Female Coinfect** |
| --- | --- | --- | --- | --- | --- | --- | --- | --- |
| **RVW** | **1354.5±148** | **1212.4±161** | **1104±228** | **1351±23** | *******  **2129±159** | **1162±99** | **#**  **1285±83** | *** 927±84** |
| **LVW** | **1694±26** | **1543±46** | **1804±87** | *****  **1383±84** | **1735±19** | *****  **1254±119** | **1850±77** | *****  **1850±31** |
| **Septal W** | **3375±95** | **3909±189** | **3555±81** | *****  **4434±170** | ******  **4001±17.3** | ******  **2828±319** | **3677±165** | *****  **2775±85** |

* p<0.05, ** p<0.01, and *** p<0.001 compared to uninfected sex matched mice (n=4/sex/group). # p ≤ 0.05 compared between coinfected and sex matched *T. cruzi* infected mice.
